# Comparing Raman Spectroscopy-Based Artificial Intelligence to High-Definition White Light Endoscopy for Endoscopic Diagnosis of Gastric Neoplasia: A Feasibility Proof-of-Concept Study

**DOI:** 10.3390/diagnostics14242839

**Published:** 2024-12-17

**Authors:** Tse Kiat Soong, Guo Wei Kim, Daryl Kai Ann Chia, Jimmy Bok Yan So, Jonathan Wei Jie Lee, Asim Shabbbir, Jeffrey Huey Yew Lum, Gwyneth Shook Ting Soon, Khek Yu Ho

**Affiliations:** 1 Department of Surgery, National University Hospital, NUHS Tower Block Level 8, 1E Kent Ridge Road, Singapore 119228, Singapore; xtaikest@gmail.com (T.K.S.); daryl_chia@nuhs.edu.sg (D.K.A.C.); sursbyj@nus.edu.sg (J.B.Y.S.); asim_shabbir@nuhs.edu.sg (A.S.); 2 Crest Surgical Practice, #08-03 Gleneagles Medical Centre, 6 Napier Rd, Singapore 258499, Singapore; jinguowei@gmail.com; 3 Department of Medicine, Yong Loo Lin School of Medicine, National University of Singapore, NUHS Tower Block Level 10, 1E Kent Ridge Road, Singapore 119228, Singapore; jonathan_wj_lee@nuhs.edu.sg; 4 Department of Pathology, National University Hospital, 1 Main Building, Level 3, 5 Lower Kent Ridge Road, Singapore 119074, Singapore; jeffrey_hy_lum@nuhs.edu.sg (J.H.Y.L.); gwyneth_st_soon@nuhs.edu.sg (G.S.T.S.)

**Keywords:** artificial intelligence, raman spectroscopy, endoscopy, gastric cancer, diagnosis

## Abstract

Background: Endoscopic assessment for the diagnosis of gastric cancer is limited by interoperator variability and lack of real-time capability. Recently, Raman spectroscopy-based artificial intelligence (AI) has been proposed as a solution to overcome these limitations. Objective: To compare the performance of the AI-enabled Raman spectroscopy with that of high-definition white light endoscopy (HD-WLE) for the risk classification of gastric lesions. Methods: This was a randomized double-arm feasibility proof-of-concept trial in which participants with suspected gastric neoplasia underwent endoscopic assessment using either the Raman spectroscopy-based AI (SPECTRA IMDx™) or HD-WLE performed by expert endoscopists. Identified lesions were classified in real time as having either low or high risk for neoplasia. Diagnostic outcomes were compared between the two groups using histopathology as the reference. Results: A total of 20 patients with 25 lesions were included in the study. SPECTRA, in real-time, performed at a statistically similar level to that of HD-WLE performed by expert endoscopists, achieving an overall sensitivity, specificity, and accuracy of 100%, 80%, and 89.0%, respectively, by patient; and 100%, 80%, and 92%, respectively, by lesion, while expert endoscopists using HD-WLE attained a sensitivity, specificity, and accuracy of 100%, 80%, and 90%, respectively, by patient; and 100%, 83.3%, and 91.7%, respectively, by lesion, in differentiating high-risk from low-risk gastric lesions. Conclusions: The SPECTRA’s comparable performance with that of HD-WLE suggests that it can potentially be a valuable adjunct for less experienced endoscopists to attain accurate and real-time diagnoses of gastric lesions. Larger-scale prospective randomized trials are recommended to validate these promising results further.

## 1. Introduction

While white light endoscopy (WLE) remains the gold standard for the diagnosis of gastric cancer, it is limited by the fact that endoscopists are only able to characterize lesions based on gross morphological changes in the gastric mucosa. Precise recognition of the latter is dependent on operator experience and is subject to interoperator variability [1]. Thus, further confirmation of the lesion often requires random biopsies, which may prove to be inefficient and be subject to sampling errors [2]. There is therefore an unmet need for a real-time endoscopic diagnostic tool that can facilitate onsite decision-making.

In recent years, endoscopic artificial intelligence (AI) has emerged as a means to reduce interoperator variability [3]. As its diagnostic performance is mostly based upon rapid interpretation of endoscopic images, it is limited by the need for high-quality images [4]. Low-quality images, which are not uncommonly encountered in real life, may lead to over-sensing and many false positives [5]. To overcome this limitation, a non-image-based AI, such as that based upon Raman spectroscopy, has been proposed as a possible solution [6].

Raman spectroscopy involves the interaction of light with molecular vibrations within a given tissue, resulting in a shift in the laser photon energy, creating a unique spectral fingerprint that reflects the molecular constituents in the tissue. This can be harnessed to differentiate molecularly altered diseased tissues from normal ones [7]. Integrating Raman spectroscopy with endoscopy allows endoscopists to obtain molecular information about suspicious lesions [8]. As biochemical constituents in the mucosa change progressively during neoplastic transformation, the molecular characteristics can also be used to determine tissue progression to neoplasia [9]. Raman spectroscopy offers additional advantages as an endoscopic diagnostic tool. It is a non-destructive method requiring minimal sample preparation. There is no contrast reagent required for its use in identifying both micro- and macro-molecules. [7] The technology could also be augmented by machine learning [6]. Once the identification algorithm has been fine-tuned, it could serve as an extremely powerful adjunct in endoscopic diagnostics.

Previous studies from our center have evaluated the use of Raman spectroscopy to diagnose gastric lesions. Although those studies were conducted in vivo in human subjects, they collected spectral data using a Raman spectroscopy research prototype that had to be analyzed post hoc. The post hoc analysis results showed Raman endoscopy could diagnose gastric dysplasia with high sensitivity, specificity, and accuracy of up to 94.4%, 96.3%, and 80.0%, respectively [9,10]. The post hoc diagnostic performance of the Raman spectroscopy research prototype system in other gastric tissues was also high, achieving sensitivity and specificity in normal mucosa, intestinal metaplasia, and early gastric cancer of 75.9% and 87.2%; 46.7% and 87.6%; and 83.3% and 95.8%, respectively [11,12]. Another group combined Raman spectroscopy with machine learning and used it to evaluate ex vivo surgical specimens. Their Raman spectroscopy-AI system was able to achieve an accuracy of 96.2% in diagnosing early gastric cancer [13]. Little has been published on the diagnostic performance of Raman spectroscopy-based AI versus WLE in the real-life evaluation of gastric lesions.

The present study was motivated by the unmet need for an endoscopic real-time diagnostic solution that does not rely upon image interpretation. Existing endoscopic artificial intelligence systems, which are dependent upon high image quality, are not able to obviate this need.

The aim of the current feasibility proof of concept study was therefore to compare the performance of the AI-enabled Raman spectroscopy system with that of HD-WLE for the risk classification of endoscopically suspected gastric lesions, using histopathologic diagnosis as the reference gold standard. It was intended to provide preliminary data so that a bigger study could be planned and conducted in the near future.

We believe the proposed study would make a significant contribution to literature in the following aspects:

- Unlike previous Raman spectroscopy-related publications that presented data based upon post hoc analysis [7,9,10,11,12,13], the proposed study is unique in that it aimed to evaluate the use of Raman spectroscopy-AI to diagnose gastric lesions in real time. In other words, the Raman spectroscopy-AI would perform the spectral analysis in real time and display the diagnosis in real time.
- This was possible because, unlike previous publications that used research prototypes [7,9,10,11,12,13], the proposed study aimed to use a product that was already approved by the Health Sciences Authority of Singapore.
- Unlike previous publications, which were mostly focused on using cohort design, this study would employ a randomized double-arm design.

## 2. Material and Methods

### 2.1. Study Design and Setting

This was a randomized double-arm prospective trial conducted at the National University Hospital, Singapore, between August 2020 and July 2022, during the pandemic period. The trial involved the endoscopic assessment of endoscopically suspected precancerous and neoplastic gastric lesions using the AI-enabled Raman spectroscopy system (experimental arm) or high-definition WLE (HD-WLE) (standard-of-care arm) and aimed to compare the diagnostic performance of the two modalities, using histopathologic diagnosis as the gold standard.

### 2.2. Ethical Considerations

The clinical trial protocol, developed following the ethical principles of the Declaration of Helsinki, was reviewed and approved by the institutional Domain-specific Review Board (Reference No: 2020/00822). This trial was conducted according to the Good Clinical Practice guidelines of the International Council for Harmonisation.

### 2.3. Study Participants and Eligibility Criteria

Participants were recruited from patients who underwent esophagogastroduodenoscopy (EGD) at the Endoscopy Centre, National University Hospital, Singapore. Patients were enrolled if they met the following criteria: aged 21 years and above; and undergoing EGD for suspected intraepithelial neoplasia or gastric cancer or known history of histologically proven intestinal metaplasia or for follow-up surveillance of the stomach after previous resection for intraepithelial neoplasia/gastric cancer. Pregnant women and patients with advanced gastric cancer based on clinical staging (>cT1 tumors), coagulopathies, active bleeding, rare malignancies (gastrointestinal stromal tumors, lymphoma, and neuroendocrine tumors), and severe life-limiting co-morbid illnesses were excluded. After explaining the study procedures, potential risks and benefits, and the requirements for participation to prospective participants, written informed consent to participate was obtained from all subjects involved in the study. Recruited patients were then screened, and those who met the eligibility criteria were enrolled in the trial. Patients who did not meet the eligibility criteria were excluded prior to the randomization as detailed in Section 2.4.1.

### 2.4. Trial Procedures

#### 2.4.1. Randomization and Masking

Participants who met the inclusion and exclusion criteria were recruited consecutively. They were randomly assigned in a 1:1 ratio to undergo endoscopic assessment using the SPECTRA IMDx™ (Endofotonics Pte Ltd., Singapore) or HD-WLE. Randomization was based on random permuted blocks, assuming equal allocation between the two study arms. This was achieved by means of a web-based randomization program (rand.scri.edu.sg) provided by the Singapore Clinical Research Institute.

The pathologists performing the histopathologic examinations of biopsied tissue specimens were blinded to the randomization of the participants. In addition, to prevent ascertainment bias, pathologists were also blinded to the endoscopic diagnoses made using the SPECTRA IMDx™ (Endofotonics Pte Ltd., Singapore) or HD-WLE. They were unblinded only at the end of the trial. The study participants, endoscopists, and the research nurse were, however, not masked from the participants’ assignment to the endoscopic assessment arms.

#### 2.4.2. Endoscopy

Based on the randomization, the study’s endoscopists then inspected the gastric mucosa and examined in detail any suspicious lesion using either the HD-WLE or the SPECTRA IMDx™ to make a diagnosis. The endoscope used for HD-WLE was either the Fujifilm VP-7000 (Fujifilm Corporation, Tokyo, Japan) or the Olympus CV-290 (Olympus Corporation, Tokyo, Japan). The endoscopists (GWK, JBYS, JWJL) were senior endoscopists with many years of experience in endoscopy and had been specifically trained in the use of the SPECTRA IMDx™. Prior to the study, they were also trained to characterize gastric lesions endoscopically using videos and an e-learning system [14]. During the endoscopic examination, a research nurse accompanying the attending endoscopist noted the findings. The endoscopic examination was conducted according to the protocol proposed by Yao [15]. Suspicious lesions detected were mapped by location to the following gastric sites, namely, the antrum (lesser curve), antrum (greater curve), anterior antrum, posterior antrum, body (lesser curve), body (greater curve), anterior body, posterior body, incisura, fundus, cardia (lesser curve), cardia (greater curve), anterior cardia, and posterior cardia.

#### 2.4.3. SPECTRA IMDx™

**Figure 1:**
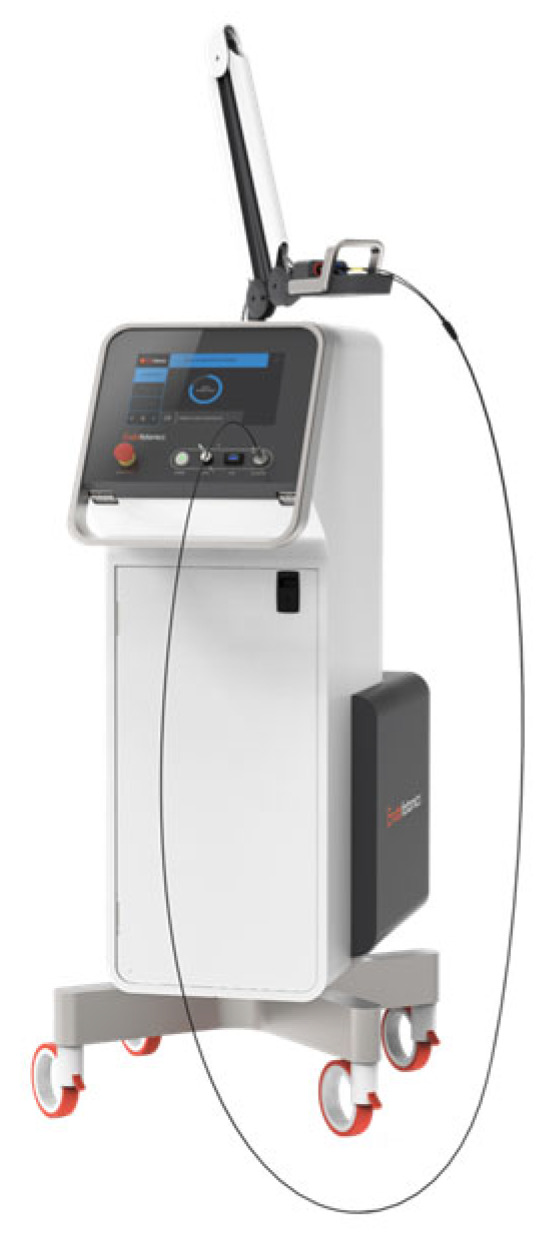
The SPECTRA IMDx™ system.

The SPECTRA IMDx™ (Endofotonics Pte Ltd., Singapore) is an AI-enabled Raman spectroscopic system that can be adapted to any standard endoscope (Figure 1). It comes with a laser system, a spectrometer, a computer with an analytical algorithm installed, and a self-engineered optical probe [16]. When in use, the laser system emits a 785 nm near-infrared laser that is transmitted through the 2 m long and 2 mm wide optical probe. The probe can be passed through the 2.8 mm wide working channel of the endoscope to interrogate the target tissue. The laser power output at the distal end of the probe and acquisition time were set at 50 milliwatts and 100 milliseconds, respectively. When the laser is interrogated upon the gastric mucosal surface, it appears as a 2 mm light spot. The light energy is absorbed by the gastric mucosa and then reflected. The resultant Raman scattering is captured by the distal end of the optical probe, transmitted back to the main system, and passed through the spectrometer. The collected signal is then processed to a clearer Raman signal. To ensure optimal signal collection, the distal end of the optical probe can be cleaned with saline flushed through the working channel of the endoscope. Diagnosis is based on the unique biomolecular spectroscopic fingerprint obtained, which is used to characterize the tissue, discriminating neoplastic tissue from normal ones based on known molecular changes associated with the development of gastric neoplasia [16].

**Figure 2:**
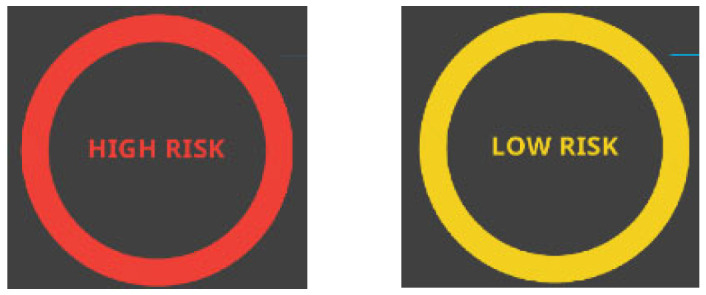
Visual output displayed on the SPECTRA IMDx™’s user interface.

An in-built AI algorithm then analyzed the acquired Raman fingerprints and risk-stratified them based on the predicted risk for neoplasia (either as high risk or low risk), the result of which is flashed on the SPECTRA IMDx™’s user interface in real-time (Figure 2). The entire process takes about 3 to 5 s.

#### 2.4.4. Tissue Biopsy and Histopathologic Examination

**Figure 3:**
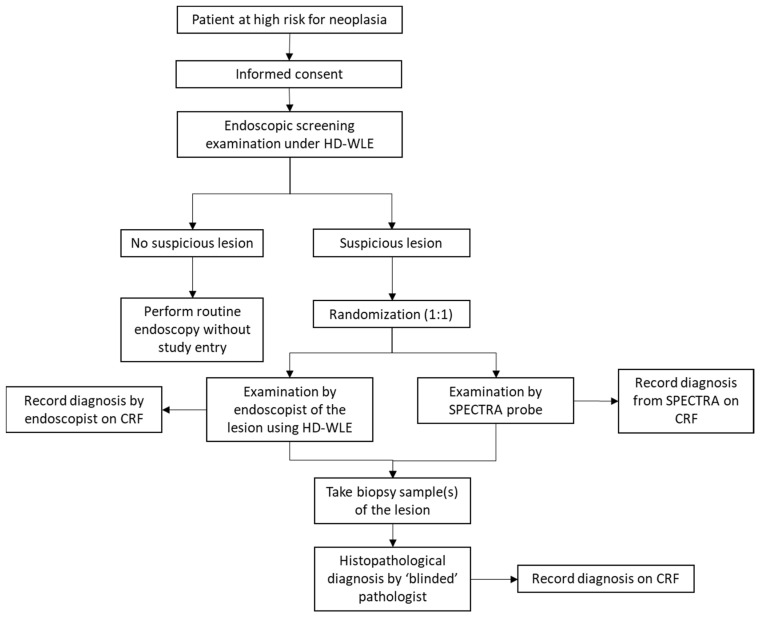
Study workflow (case report form, CRF).

For all lesions examined endoscopically, whether by HD-WLE or SPECTRA IMDx™, one or more biopsies were obtained from the sites of each suspected lesion. Mucosal biopsies were only obtained from the sites of endoscopically suspected lesions and not from endoscopically normal-appearing tissue. The number of biopsy samples was determined by the lesion size. In general, if the lesion was smaller than 1 cm, only one biopsy sample was taken from the center of the lesion. If the lesion was 1–2 cm, two biopsies were taken from the center and edge of the lesion. One more biopsy sample was taken for every subsequent 1 cm size increment from the center and edges of the lesion. The reason for the increased number of biopsies was to ensure the biopsies were representative of the lesion’s histopathology. Mucosal biopsies were obtained at the exact sites where the SPECTRA IMDx probe had been placed. The acquired specimens were sent to the assigned gastrointestinal pathologist (JHYL) for examination using standard histopathologic techniques. The histopathological examination of gastric cancer followed the WHO Classification and Lauren Classification system of gastric cancer [17]. To ensure internal consistency in the diagnosis across biopsies, a second pathologist (GSTS) independently examined each biopsy specimen, and a consensus on the diagnosis was sought if there was a disagreement in the diagnosis. The study workflow is depicted in Figure 3.

### 2.5. Study Endpoints

The endpoint of this study is a diagnosis made by the endoscopist through either the SPECTRA or HD-WLE, with reference to the histopathologic examination results.

#### 2.5.1. Endoscopic Diagnosis and Classification of Gastric Lesions

For the purpose of this study, all lesions were classified as either “high risk of neoplasia” or “low risk of neoplasia”, with high-grade intraepithelial neoplasm (HGIN) and gastric cancer being classified as “high risk of neoplasia” while other findings such as gastritis, intestinal metaplasia, low-grade intraepithelial neoplasm (LGIN), and other non-cancerous lesions being classified as “low risk of neoplasia”. The classification was based on treatment outcomes since HGIN, and cancer is generally treated with resection, while low-risk lesions can be closely followed up or discharged [18]. Under the SPECTRA IMDx™ investigation arm, lesions detected by the system were automatically categorized by the in-built AI algorithm as having a “low risk of neoplasia” or a “high risk of neoplasia” in real time (Figure 2). The Raman diagnosis was prospectively recorded in the case report form (CRF) by the research nurse. Under the HD-WLE investigation arm, endoscopists described each visually detected lesion in terms of its size, morphology, color, characteristics, and boundaries and then classified it as either “high risk” or “low risk of neoplasia” in real time. The endoscopic diagnosis was also prospectively recorded in the CRF by the research nurse (Figure 3).

#### 2.5.2. Outcome Measures

The primary outcome of this study was the real-time performance metrics of the Raman spectroscopy-AI and WLE in comparison to the diagnosis obtained from the histopathologic examination of the corresponding biopsy specimen, which is the present gold standard for diagnosis of early and pre-malignant gastric lesions. The primary outcome measures included the real-time diagnostic sensitivity, specificity, and accuracy of the respective modality (SPECTRA IMDx™ or HD-WLE) to differentiate the presence or absence of high-risk neoplastic lesions. The secondary outcome measures include the respective modality’s positive predictive value (PPV) and negative predictive value (NPV).

### 2.6. Statistical Analysis

As this was an exploratory study, the number of subjects included in the analysis was not based on statistical power calculation. The sample size was based on what was pragmatically feasible during the pandemic period. We believe this pragmatic approach was acceptable for a feasibility proof-of-concept study of this nature.

The real-time diagnostic accuracy, sensitivity, specificity, PPV, and NPV were calculated for both the SPECTRA IMDx™ and HD-WLE. The diagnostic accuracy between the SPECTRA and HD-WLE was computed via R for Microsoft Excel (version 4.3.2). Fisher’s Exact Test was performed to compare the diagnostic metrics via R.

## 3. Results

### 3.1. Study Participants

A total of 21 patients were enrolled in the study. Of the 21 participants, 11 were randomized to the HD-WLE arm and 10 to the SPECTRA arm. The mean age of the entire population was 71.7 years old, with 12 (57.1%) being male participants. Nineteen (90.5%) of participants were Chinese. Ten (47.6%) of participants had prior gastric lesions. The demographics of the participants, stratified by the interventional arm, are summarized in Table 1. There was no statistical difference in the demographics and clinical characteristics between the two groups.

### 3.2. Diagnostic Outcomes

One patient from the HD-WLE group was excluded since he had no lesion found during his endoscopic examination. In the remaining 20 patients (10 in the HD-WLE arm and 10 in the SPECTRA arm), 61 tissue samples (30 from the HD-WLE arm and 31 from the SPECTRA arm) were obtained from 25 lesions (12 lesions in the HD-WLE arm and 13 lesions in the SPECTRA arm) for histopathologic examination. Owing to poor Raman spectral data, a total of seven tissue samples from one of the lesions found in one of the patients in the SPECTRA arm were excluded from further analysis. Unlike other patients who underwent their SPECTRA examination in the endoscopy suites, this patient underwent the SPECTRA examination during concurrent laparoscopic examination in the operating theater. The strong laparoscopic lighting likely caused interference with the collection of the Raman signal. Exclusion of this patient and his data brought the total number of patients included in the SPECTRA arm to nine, with a total of 24 sampled points from 12 sampled lesions. Thus, 12 lesions from 10 patients in the HD-WLE arm were compared with 12 lesions from 9 patients in the SPECTRA arm in the final per patient and per lesion analysis.

#### 3.2.1. Outcomes by Patient

As shown in Table 2, both HD-WLE and SPECTRA performed very well in real-time diagnosing high- and low-risk neoplastic lesions in each patient when using histology as the gold standard. The per-patient sensitivity, specificity, PPV, NPV, and accuracy of HD-WLE in real-time differentiating high- from low-risk neoplastic lesions were 100% (95% CI: 48–100%), 80.0% (95% CI: 28–99%), 83.3% (95% CI: 36–100%), 100% (95% CI: 40–100%), and 90.0% (95% CI: 55–100%), respectively. These numbers were statistically similar to the per-patient sensitivity, specificity, PPV, NPV, and accuracy of SPECTRA in real-time classifying high-risk and low-risk neoplastic lesions of 100% (95% CI: 40–100%), 80.0% (95% CI: 28–99%), 80.0% (95% CI: 28–99%), 100% (95% CI: 40–100%), and 90.0% (95% CI: 52–100%), respectively (*p* = 1.00).

#### 3.2.2. Outcomes by Lesion

Lesion-level analysis also showed that both HD-WLE and SPECTRA were equally as good in real-time diagnosing high-risk and low-risk gastric neoplastic lesions when compared with histology. When examining the 12 lesions, HD-WLE yielded a sensitivity, specificity, PPV, NPV, and accuracy of 100% (95% CI: 54–100%), 83.3% (95% CI: 36–100%), 85.7% (95% CI: 42–100%), 100% (95% CI: 48–100%), and 91.7% (95% CI: 62–100%), respectively. In the SPECTRA arm, there were also a total of 12 lesions available for analysis. The per-lesion sensitivity, specificity, PPV, NPV, and accuracy of SPECTRA in real-time gastric risk stratification were 100% (95% CI: 59–100%), 80.0% (95% CI: 28–99%), 87.5% (95% CI: 47–100%), 100% (95% CI: 40–100%), and 92.0% (95% CI: 62–100%), respectively. Table 2 showed the detailed results.

## 4. Discussion

The study adds to a growing body of evidence that Raman spectroscopy is an emerging technology that can be applied to enhance the rapid diagnosis of gastric neoplasia [6,7,8,9]. Against this backdrop, the SPECTRA was developed to further enhance the real-time diagnostic capability of Raman spectroscopy by incorporating machine learning algorithms into the system [6]. As little had been published on the diagnostic performance of Raman spectroscopy-based AI in the real-life evaluation of gastric lesions, this feasibility proof-of-concept study provided some of the first preliminary data on its diagnostic performance in the real-world clinical setting. It showed that the Raman spectroscopy-based AI system (SPECTRA IMDx™) was able to differentiate high-risk neoplastic gastric lesions, including HGIN and cancer, from low-risk gastric lesions, including gastritis, intestinal metaplasia, and LGIN, with high sensitivity, specificity, and accuracy, using histopathologic diagnosis as the reference gold standard.

Although most studies prefer per-subject analysis over per-lesion analysis [19], both analyses were performed in this study and showed similarly good diagnostic outcomes for both the HD-WLE and SPECTRA arms per patient and per lesion. The consistent results add to the strength of this study’s two notable findings. Firstly, HD-WLE, when used by experienced endoscopists, was able to risk-stratify gastric lesions with high performance indices. On first impression, the >80% sensitivity, specificity, and accuracy results achieved by the HD-WLE cohort could be conceived as being too good. However, supporting this finding was a recent publication by the same group, which demonstrated the endoscopists were able to evaluate endoscopic images with high accuracy, sensitivity, and specificity of 0.847, 0.525, and 0.872, respectively [20]. Thus, we believe the superior diagnostic performance of HD-WLE found in this study is real and is due to the endoscopists’ superior training and experience working in a tertiary center in Singapore. The second notable finding from this study is that SPECTRA was equally as good as HD-WLE used by experienced endoscopists in risk stratifying gastric lesions. This is a positive finding, as it means that this AI system will be useful to augment endoscopists of different abilities and not just those coming from tertiary centers. Furthermore, the risk stratification by SPECTRA was conducted in real-time, with its readings displayed and recorded prospectively. Albeit this being a preliminary result, the 92% diagnostic accuracy rate suggested the SPECTRA AI system was mature and should be able to support real-time decision-making strategies in the near future.

Besides SPECTRA’s ability to address the issue of interoperator variability, it has other advantages. In a recent study comparing the performance of three endoscopy AI polyp detection systems, the per-frame sensitivities were found to vary between the different systems [21]. The inter-system variability is inherent in image-based endoscopy AI systems. Being a non-image-based AI system, SPECTRA does not require images for its training, analysis, and diagnostics. Thus, it does not suffer from the “garbage in garbage out” issue [4]. Busy centers, where acquired images are not always “perfect”, may find non-image-based AI systems more suitable for their needs. Furthermore, a previous report suggested that sole reliance on gross morphological appearance during endoscopy was inadequate to accurately diagnose cervical lesions [22]. The addition of data in the form of biochemical information, which can be provided by Raman spectroscopy [23], will be important to strengthen the AI diagnosis. Lastly, Raman spectroscopy [24], as used in this study, is label-free. No tissue damage was incurred for data acquisition during the procedure.

While the above results are promising, there are some limitations in the interpretation of the findings from this study that we would like to highlight. Firstly, the study was limited by the small number of participants, consistent with the feasibility design of the study. Ideally, we would have liked to evaluate this diagnostic method in a larger subject group. However, this was not possible as the study was conducted during the pandemic period, and recruitment of subjects was extremely challenging during that extraordinary time. Being a non-COVID-related study, it was also suspended at one stage, in accordance with the institutional policy. We decided to complete the analysis after the pandemic period, as we felt the findings were important and could still contribute immensely to scientific literature. Furthermore, this study was intended to provide feasibility data so that a bigger study could be conducted in the future. The limited sample size would not detract from the study aim and would still provide valid findings for a feasibility proof-of-concept study of this nature.

While the duration and number of participants were not ideal, the randomized and prospective nature of the study and the robust histologic validation process still lend credibility to prove the feasibility of using Raman spectroscopy-AI as an adjunct to HD-WLE. Moreover, several data points had to be excluded from the analysis owing to various factors, such as interfering signals from light contamination and poor probe contact. These resulted in data points that provided weak signals, noisy signals, and contaminated data that were not representative of tissue. However, such loss of data points can be minimized with increasing experience in the use of the equipment and with possible modifications to the hardware and software processing to reduce external disturbances and improve the quality of data obtained in the future, thereby enhancing its diagnostic accuracy.

While there are limitations as described above, the nature of the software program makes it amenable to further augmentation via AI and machine learning, thus giving the technology boundless potential for growth and improvement in its diagnostic accuracy. With improvements to the technology, it could serve to aid decision-making in whether to take biopsies of borderline lesions, the best site to take biopsies, in prediction of the risk of lesions, and to guide endoscopists to determine the margin of resection.

In this study, we did not compare the times to complete the diagnosis between the two arms. However, we do not think the absence of comparison data would diminish the scientific value of our manuscript, as both diagnostic processes did not significantly prolong the duration of the endoscopic examination. Typically, the endoscopic examination took 10–20 min to complete depending on the number of biopsies required. In the HD-WLE arm, the addition of a careful endoscopic examination and documentation of the findings added an extra 1–2 min to the workflow. In contrast, the use of the Raman probe increased the examination duration by approximately 2–3 min, since typically all the sites were interrogated by the Raman probe sequentially before it was removed from the biopsy channel of the endoscope. We would like to emphasize that SPECTRA IMDx’s time-saving benefit is greatest if multiple biopsies are desired, as taking each mucosal biopsy requires the biopsy forceps to be retrieved, the tissue deposited into the specimen jar, and the biopsy forceps re-introduced through the channel of the endoscope to reach the target. In contrast, the Raman probe can be left in the working channel while multiple sites are being interrogated, resulting in tremendous time savings.

Lastly, although this study has suggested the feasibility of deploying Raman spectroscopy-AI in the clinical setting, certain issues will need to be addressed before the technology can gain widespread clinical adoption. A previous review suggested that clinical safety, efficacy, and cost-effectiveness must be demonstrated before a technologic solution can be adopted into clinical practice [25]. In this regard, a Markov modeling study from Europe showed that AI decreased the costs per colonoscopy screened individual from 3400 USD to 3343 USD, suggesting that the addition of AI into screening colonoscopy is a cost-saving strategy to prevent colorectal cancer incidence and mortality [26]. The issue of cost-effectiveness of an AI system in diagnosing early gastric cancers in Japan was also assessed using a Markov model. The study showed that AI was cost-effective if the device cost less than 104 USD [27]. Thus, limited data seems to suggest AI can be a cost-effective diagnostic adjunct to gastrointestinal endoscopy. The current SPECTRA IMDx™ was designed to classify gastric lesions into high-risk and low-risk categories, which served to support the endoscopists’ decisions of whether to resect a lesion. It can be argued that such a risk classification may not be good enough for those endoscopists who wish to make an exact diagnosis of gastric lesions, including the types of dysplasia or cancer. While having a Raman spectroscopy-AI with an expanded diagnostic capability would be ideal, developing such an AI system will entail a much larger training and validation dataset. Ultimately, the issue is whether an expanded diagnostic capability will improve clinical outcomes, thus justifying the increased resources needed for its development.

## 5. Conclusions

The SPECTRA performed at a similarly high accuracy as expert endoscopists using HD-WLE, suggesting a use case in which non-expert endoscopists can improve patient outcomes via more accurate and real-time diagnosis of high-risk lesions. The study has achieved its intended goal of providing preliminary data to show the feasibility of using Raman spectroscopy-based AI in the real-world setting. Due to the limited sample size, findings from this study should be considered preliminary and not yet generalizable to the general population. More randomized controlled trial studies with larger population sizes would be helpful to evaluate the technology further so that it could eventually be adopted into mainstream practice.

## Figures and Tables

**Table 1:** Demographic and clinical characteristics of patients included in the study.

	HD-WLE (*n* = 11)		SPECTRA (*n* = 10)		*p*
Age (mean (SD))	74.6	(9.7)	69.8	(8.2)	0.243
Gender					0.85
Male (%)	7	(63.6)	5	(50.0)	
Race					0.366
Chinese (%)	10	(90.9)	9 (90.0)	(90.0)	
Indian (%)	0	(0.0)	1 (10.0)	(10.0)	
Malay (%)	1	(9.1)	0 (0.0)	(0.0)	
Prior Gastric Lesions					0.384
Yes (%)	5	(45.5)	5	(50.0)	
Malignant (%)	3	(27.3)	1	(10.0)	
Pre-Malignant (%)	2	(18.2)	2	(20.0)	
Non-Malignant (%)	0	(0.0)	2	(20.0)	
No (%)	6	(54.5)	5	(50.0)	

**Table 2:** Diagnostic performance of HD-WLE vs. SPECTRA IMDx™ by patient and by lesion.

	Characteristics	HD-WLE	SPECTRA	*p*-Value
By patient	Total # Recruited	10	9	
	True Positive	5	4	
	True Negative	4	4	
	False Positive	1	1	
	False Negative	0	0	
	Sensitivity	100% (95% CI [48–100%])	100% (95% CI [40–100%])	1.00
	Specificity	80.0% (95% CI [28–99%])	80.0% (95% CI [28–99%])	1.00
	PPV	83.3% (95% CI [36–100%])	80.0% (95% CI [28–99%])	
	NPV	100% (95% CI [40–100%])	100% (95% CI [40–100%])	
	Accuracy	90.0% (95% CI [55–100%])	89.0% (95% CI [52–100%])	1.00
By lesion	Total # Recruited	12	12	
	True Positive	6	7	
	True Negative	5	4	
	False Positive	1	1	
	False Negative	0	0	
	Sensitivity	100% (95% CI [54–100%])	100% (95% CI [59–100%])	1.00
	Specificity	83.3% (95% CI [36–100%])	80.0% (95% CI [28–99%])	1.00
	PPV	85.7% (95% CI [42–100%])	87.5% (95% CI [47–100%])	
	NPV	100% (95% CI [48–100%])	100% (95% CI [40–100%])	
	Accuracy	91.7% (95% CI [62–100%])	92.0% [95% CI [62–100%]]	1.00

## Data Availability

The original contributions presented in this study are included in the article. Further inquiries can be directed to the corresponding author.

## References

[1] Yalamarthi S., Witherspoon P., McCole D., Auld C.D. (2004). Missed diagnoses in patients with upper gastrointestinal cancers. Endoscopy.

[2] Song Y.H., Xu L.D., Xing M.X., Li K.K., Xiao X.G., Zhang Y., Li L., Xiao Y.J., Qu Y.L., Wu H.L. (2021). Comparison of white-light endoscopy, optical-enhanced and acetic-acid magnifying endoscopy for detecting gastric intestinal metaplasia: A randomized trial. World J. Clin. Cases.

[3] Yu H., Singh R., Shin S.H., Ho K.Y. (2021). Artificial intelligence in upper GI endoscopy—Current status, challenges and future promise. J. Gastroenterol. Hepatol..

[4] Almadi M.A., Ho K.Y. (2020). Artificial inelegance in endoscopy: An updated auricle of Delphi!. Saudi J. Gastroenterol..

[5] Mori Y., Bretthauer M. (2021). Addressing false-positive findings with artificial intelligence for polyp detection. Endoscopy.

[6] Ho K.Y. (2022). Beyond images: Emerging role of Raman spectroscopy-based artificial intelligence in diagnosis of gastric neoplasia. Chin. J. Cancer Res..

[7] Eberhardt K., Stiebing C., Matthäus C., Schmitt M., Popp J. (2015). Advantages and limitations of Raman spectroscopy for molecular diagnostics: An update. Expert Rev. Mol. Diagn..

[8] Sharma N., Takeshita N., Ho K.Y. (2016). Raman Spectroscopy for the Endoscopic Diagnosis of Esophageal, Gastric, and Colonic Diseases. Clin. Endosc..

[9] Huang Z., Teh S.K., Zheng W., Lin K., Ho K.Y., Teh M., Yeoh K.G. (2010). In vivo detection of epithelial neoplasia in the stomach using image-guided Raman endoscopy. Biosens. Bioelectron..

[10] Huang Z., Bergholt M.S., Zheng W., Lin K., Ho K.Y., Teh M., Yeoh K.G. (2010). In vivo early diagnosis of gastric dysplasia using narrow-band image-guided Raman endoscopy. J. Biomed. Opt..

[11] Bergholt M.S., Lin K., Wang J., Zheng W., Xu H., Huang Q., Ren J.L., Ho K.Y., Teh M., Srivastava S. (2016). Simultaneous fingerprint and high-wavenumber fiber-optic Raman spectroscopy enhances real-time in vivo diagnosis of adenomatous polyps during colonoscopy. J. Biophotonics.

[12] Bergholt M.S., Zheng W., Ho K.Y., Teh M., Yeoh K.G., So J.B., Shabbir A., Huang Z. (2013). Fiber-optic Raman spectroscopy probes gastric carcinogenesis in vivo at endoscopy. J. Biophotonics.

[13] Li C., Liu S., Zhang Q., Wan D., Shen R., Wang Z., Li Y., Hu B. (2023). Combining Raman spectroscopy and machine learning to assist early diagnosis of gastric cancer. Spectrochim. Acta. Part A Mol. Biomol. Spectrosc..

[14] Yao K., Yao T., Uedo N., Doyama H., Ishikawa H., Nimura S., Takahashi Y. (2024). E-learning system to improve the endoscopic diagnosis of early gastric cancer. Clin. Endosc..

[15] Yao K. (2013). The endoscopic diagnosis of early gastric cancer. Ann. Gastroenterol..

[16] Wang J., Lin K., Zheng W., Ho K.Y., Teh M., Yeoh K.G., Huang Z. (2016). Fiber-optic Raman spectroscopy for in vivo diagnosis of gastric dysplasia. Faraday Discuss..

[17] Kushima R. (2022). The updated WHO classification of digestive system tumours-gastric adenocarcinoma and dysplasia. Der. Pathol..

[18] Waddingham W., Nieuwenburg S.A.V., Carlson S., Rodriguez-Justo M., Spaander M., Kuipers E.J., Jansen M., Graham D.G., Banks M. (2020). Recent advances in the detection and management of early gastric cancer and its precursors. Frontline Gastroenterol..

[19] Chang K.C., Leung C.C., Tam C.M. (2006). Per lesion analysis is misleading. Thorax.

[20] Quek S.X.Z., Lee J.W.J., Feng Z., Soh M.M., Tokano M., Guan Y.K., So J.B.Y., Tada T., Koh C.J. (2023). Comparing artificial intelligence to humans for endoscopic diagnosis of gastric neoplasia: An external validation study. J. Gastroenterol. Hepatol..

[21] Troya J., Sudarevic B., Krenzer A., Banck M., Brand M., Walter B.M., Puppe F., Zoller W.G., Meining A., Hann A. (2024). Direct comparison of multiple computer-aided polyp detection systems. Endoscopy.

[22] Mirabal Y.N., Chang S.K., Atkinson E.N., Malpica A., Follen M., Richards-Kortum R. (2002). Reflectance spectroscopy for in vivo detection of cervical precancer. J. Biomed. Opt..

[23] Zavaleta C.L., Garai E., Liu J.T., Sensarn S., Mandella M.J., Van de Sompel D., Friedland S., Van Dam J., Contag C.H., Gambhir S.S. (2013). A Raman-based endoscopic strategy for multiplexed molecular imaging. Proc. Natl. Acad. Sci. USA.

[24] Liu Z., Su W., Ao J., Wang M., Jiang Q., He J., Gao H., Lei S., Nie J., Yan X. (2022). Instant diagnosis of gastroscopic biopsy via deep-learned single-shot femtosecond stimulated Raman histology. Nat. Commun..

[25] Kaan H.L., Ho K.Y. (2020). Clinical adoption of robotics in endoscopy: Challenges and solutions. JGH Open.

[26] Areia M., Mori Y., Correale L., Repici A., Bretthauer M., Sharma P., Taveira F., Spadaccini M., Antonelli G., Ebigbo A. (2022). Cost-effectiveness of artificial intelligence for screening colonoscopy: A modelling study. Lancet Digit. Health.

[27] Yonazu S., Ozawa T., Nakanishi T., Ochiai K., Shibata J., Osawa H., Hirasawa T., Kato Y., Tajiri H., Tada T. (2023). Cost-effectiveness analysis of the artificial intelligence diagnosis support system for early gastric cancers. DEN Open.

